# Girl in the cellar: a repeated cross-sectional investigation of belief in conspiracy theories about the kidnapping of Natascha Kampusch

**DOI:** 10.3389/fpsyg.2013.00297

**Published:** 2013-05-24

**Authors:** Stefan Stieger, Nora Gumhalter, Ulrich S. Tran, Martin Voracek, Viren Swami

**Affiliations:** ^1^Department of Basic Psychological Research and Research Methods, School of Psychology, University of ViennaVienna, Austria; ^2^Department of Psychology, University of WestminsterLondon, UK; ^3^Department of Psychology, HELP University CollegeKuala Lumpur, Malaysia

**Keywords:** conspiracy theories, Natascha Kampusch, repeated cross-sectional survey, individual differences, media exposure

## Abstract

The present study utilized a repeated cross-sectional survey design to examine belief in conspiracy theories about the abduction of Natascha Kampusch. At two time points (October 2009 and October 2011), participants drawn from independent cross-sections of the Austrian population (Time Point 1, *N* = 281; Time Point 2, *N* = 277) completed a novel measure of belief in conspiracy theories concerning the abduction of Kampusch, as well as measures of general conspiracist ideation, self-esteem, paranormal and superstitious beliefs, cognitive ability, and media exposure to the Kampusch case. Results indicated that although belief in the Kampusch conspiracy theory declined between testing periods, the effect size of the difference was small. In addition, belief in the Kampusch conspiracy theory was significantly predicted by general conspiracist ideation at both time points. The need to conduct further longitudinal tests of conspiracist ideation is emphasized in conclusion.

Conspiracy theories refer to explanations of important events, such as war, natural disasters, and poverty, which include “appeals to the intentional deception and manipulation of those involved in, affected by, or witnessing these events” (Basham, 2003, p. 91). Typically, these deceptions are believed to involve multiple actors working together toward some malevolent end (Barkun, 2003; Bale, 2007; Swami and Furnham, 2013). In this sense, conspiracy theorizing can be said to adopt “a counter-discourse of some sort” (Gray, 2010, p. 29); that is, conspiracy theories present a stance that is counter-hegemonic and that is opposed to orthodox, official, or received explanations of an event (Buenting and Taylor, 2010; Swami and Coles, 2010).

Traditionally, academic investigations have tended to view conspiracy theorizing as the result of pathology, both on the grounds of morality and rationality (e.g., Hofstadter, 1966; Donskis, 1998; Darwin et al., 2011; Swami et al., 2013). Such perspectives include the implicit suggestion that conspiracist ideation fosters dysfunctionality and, therefore, stunts any form of agency on the part of conspiracist actors (e.g., Quimby, 1993; Thomas and Quinn, 1993). More recently, however, a growing body of work has argued that such a lens of psychopathology is deficient (Swami and Coles, 2010; Swami and Furnham, 2013), as it fails to recognize the way in which ordinary actors use conspiracy theories to construct complex arguments in relation to intergroup relations and to challenge political, ideological, and social hegemony (Sapountzis and Condor, 2013).

Based on this perspective, a growing body of work has focused on individual difference traits that may predispose some individuals to endorse conspiracy theories. An important outcome of this work is the suggestion that conspiracy theories form part of a “monological belief system,” in which the adoption of a conspiracist worldview eases the assimilation of new conspiracy theories (Goertzel, 1994). Certainly, there is growing evidence to suggest that a stronger conspiracist worldview is associated with greater belief in specific and newly-emerging conspiracy theories (Swami et al., 2010, 2011), more conspiracist explanations for a given event (Swami and Furnham, 2012), and even adoption of contradictory explanations for the same event (Wood et al., 2012).

In tandem with this research, a small body of work has focused on the individual difference variables that are associated with conspiracist ideation. Thus, the available evidence suggests that stronger belief in conspiracy theories is associated with lower self-esteem, feelings of powerlessness and anomie, political cynicism and distrust, greater support for democratic principles, greater openness to experience, and stronger New Age beliefs (e.g., Abalakina-Paap et al., 1999; Swami et al., 2010, 2011, 2012, 2013; Darwin et al., 2011; Newheiser et al., 2011; Swami, 2012; Swami and Furnham, 2012; Jolley and Douglas, 2013). There is also some evidence that conspiracist ideation is associated with paranormal and superstitious beliefs, and lower crystallized intelligence (Swami et al., 2011), although these associations tend to be relatively weak.

Although this work has been important in shaping contemporary understandings of conspiracy theories, an important gap in the literature concerns the stability of conspiracy theorizing over time. For example, although conspiracy theories in general remain popular (Swami and Furnham, 2013), individual conspiracy theories may vary across a number of dimensions, such as their prominence in the public sphere and their longevity. Indeed, almost all psychological studies on conspiracy theories to date have focused on constrained periods of time, taking a snapshot of particular conspiracy theorizing at a given point. Although cross-sectional studies have been useful, there is a need to supplement the existing database with longitudinal studies (Uscinski et al., 2011).

Certainly, there is some evidence that broad socio-political factors influence when and where conspiracy theories become viable modes of explanation. In their longitudinal assessment of published letters to the editor of the *New York Times* from 1897 to 2010, Uscinski et al. (2011) reported that international and domestic conflicts influenced when and how conspiracy theories became more prominent in the United States. More specifically, they found that conspiracy theories were more frequent and vociferous during times of elections and wars. Even so, systematic longitudinal work on conspiracy theories remains in its infancy and further prospective work may highlight important dimensions of conspiracy theorizing that have hitherto been neglected (Uscinski et al., 2011).

For example, while the extant literature has focused on stable individual differences that correlate with conspiracy theorizing, it is also possible that change (or lack of change) can occur at the level of specific beliefs. Such change might relate to a number of different aspects, including the strength, dimensionality, content, and correlates of conspiracist beliefs. For example, changes in the strength of belief in conspiracy theories may be related to contextual factors, such as the availability of conspiracist ideation concerning a given event (e.g., in terms of media exposure; cf. Swami et al., 2010). By contrast, stability in belief in a conspiracy theory may be a function of the monological nature of conspiracy theories (Goertzel, 1994) or the fact that conspiracy theories are predicted by a set of relatively stable individual difference traits (Swami and Coles, 2010; Swami and Furnham, 2013). Understanding if and when such changes occur, therefore, remains an important task for scholars.

As a contribution to the literature, then, the present study utilized a repeated cross-sectional survey design to examine belief in a specific conspiracy theory in Austria, namely what we refer to broadly as conspiracy theories concerning the kidnapping of Natascha Kampusch, an Austrian woman. Kampusch was abducted at age 10 by Wolfgang Priklopil and held captive for more than 8 years until her eventual escape in August 2006. Media attention of Kampusch has remained in the public sphere, with Kampusch giving select interviews, hosting her own talk show in mid-2008, and publishing an autobiography in September 2010. Beginning in 2006, conspiracy theories of the Kampusch kidnapping have circulated, mainly on the Internet, with various strands including the suggestion that there were multiple perpetrators of her abduction and that investigators covered up evidence.

Conspiracy theories of the Kampusch kidnapping were selected as the focus of the present study as it provides a useful snapshot of a relatively localized narrative of an event of national significance in Austria. Here, we report on data obtained at two time points—October 2009 and October 2011 (selected as matter of convenience)—and drawn from independent cross-sections of the Austrian population. The two time points represent periods when it is thought that Kampusch conspiracy theories had reached saturation in Austria. Although repeated cross-sectional evidence is considered to be inferior to true longitudinal evidence or panel data (that is, data from the same cross-sectional units over time), this nevertheless provides more information than data from a single cross-sectional survey and avoids known problems of sample attrition and response bias (Moffit, 1993; Verbeek and Vella, 2005). Given the lack of previous longitudinal work, this part of the study was largely exploratory.

In addition, we also examined the influence of individual difference factors known to shape conspiracist ideation (i.e., general conspiracist ideation, self-esteem, paranormal and superstitious beliefs, crystallized intelligence, and media exposure) on belief in the Kampusch conspiracy theories at both time points. Specifically, and based on previous work, we expected that belief in the Kampusch conspiracy theory would be associated with greater general conspiracist ideation, lower self-esteem, greater paranormal and superstitious beliefs, lower intelligence, and greater media exposure at both time points.

## Methods

### Participants

#### Time point 1

Participants in the first testing period were 169 women and 112 men recruited from among the general population in Austria. Participants ranged in age from 18 to 80 years (*M* = 33.83, *SD* = 15.44). All participants were of European Caucasian descent and, in terms of educational qualifications, 5.4% had completed minimum secondary education, 24.2% had completed an apprenticeship, 55.4% had a college diploma, 14.6% had a university degree, and the remainder had some other qualification.

#### Time point 2

At the second testing period, the participants were 184 women and 89 men (age range 16–60 years, *M* = 25.97, *SD* = 8.54). All participants were of European Caucasian ancestry and 2.6% had completed minimum secondary schooling, 6.2% had an apprenticeship, 70.8% had a college diploma, 20.4% had a university degree, and the remainder had some other qualification.

### Materials

#### Conspiracist beliefs about Natascha Kampusch

To measure conspiracist beliefs about the kidnapping of Natascha Kampusch, we purpose-designed a novel 12-item scale using standard scale-development procedures (Spector, 1992). Authors SS, NG, and MV initially reviewed the available conspiracist literature about the kidnapping of Natascha Kampusch. Based on this review, each author generated items independently, which were then discussed in a group session until consensus was reached. This process led to the final version of the Kampusch Conspiracy Scale (KCS), with items reflecting aspects of conspiracist theorizing about the kidnapping and aftermath. The 12 items on this scale, which were rated on a 9-point Likert-type scale (1 = Completely false, 9 = Completely true), are presented in Table 1. The factor structure and reliability of this scale is explored in the Results section.

**Table 1:** **Items in the Kampusch conspiracy scale and factor loadings following exploratory factor analysis**.

**Items**	**Time Point 1**	**Time Point 2**
(1) The mother of Natascha Kampusch—Mrs. Brigitta Sirny—and Wolfgang Priklopil knew each other even before the kidnapping.	0.15	0.29
(2) The fact that Natascha Kampusch was carrying her passport with her at the time of her kidnapping indicates that her mother knew about the planned kidnapping.	**0.33**	**0.30**
(3) It is implausible that Natascha Kampusch did not have an opportunity to flee during her 8-year kidnapping, as she was allowed to make phone calls and occasional outdoor trips with Priklopil.	−0.04	−0.01
(4) Herwig Haidinger—the former head of the Federal Bureau of Criminal Investigation—was actively prevented from seeing witness reports by the state attorney's office in order to follow-up failures during the investigation.	0.05	0.13
(5) Natascha Kampusch became pregnant by Priklopil during her imprisonment, but hints about this were destroyed.	0.21	0.16
(6) Although the police refute the “multiple-perpetrator-theory” in their reports, the witness report of the 12-year-old friend of Natascha Kampusch, who witnessed the kidnapping, reinforces this hypothesis.	**0.60**	**0.56**
(7) The parents of Natascha Kampusch sold her to Wolfgang Priklopil for financial gain.	**0.42**	**0.31**
(8) The Federal Office of Justice knowingly protected a child pornography ring during the investigation.	**0.60**	**0.64**
(9) The only witness, who stated having seen a second perpetrator in the car used for the kidnapping, was later forced to revise her testimony.	**0.68**	**0.73**
(10) The police paid too little attention to some evidence (e.g., an anonymous phone call), which points to a cover-up.	**0.75**	**0.76**
(11) Franz Kröll, the former chief investigator, was hindered by the Federal Office of Justice from investigating the “multiple-perpetrator-theory” more closely.	**0.48**	**0.42**
(12) Franz Kröll, the former chief investigator, was placed under such heavy pressure as a result of his investigations that he committed suicide in 2010.	**0.65**	**0.78**

Values in bold indicate that an item was retained.

Note: items were presented in a random order in the survey.

#### Conspiracist ideation

To measure general conspiracist ideation, we included the Belief in Conspiracy Theories Inventory (Swami et al., 2010, 2011; German translation: Swami et al., 2011), a 15-item measure that describes a range of internationally-recognizable conspiracy theories (sample item: “A powerful and secretive group, known as the New World Order, are planning to eventually rule the world through an autonomous world government, which would replace sovereign governments”). All items were rated on a 9-point Likert-type scale (1 = *Completely false*, 9 = *Completely true*) and an overall score was computed as the mean of all items (higher scores reflect greater belief in conspiracy theories). Previous work has shown that the scale is one-dimensional and its item pool has good internal consistency (Swami et al., 2010, 2011) and correlates strongly with scores from a non-event based, generic measure of conspiracist ideation (*r* = 0.88; Brotherton et al., 2012). In the present work, Cronbach's α for this scale was 0.87 at both time points.

#### Self-esteem

We measured self-esteem using Rosenberg's Self-Esteem Scale (Rosenberg, 1965; German translation: von Collani and Herzberg, 2003), a 10-item measure of an individual's self-worth (sample item: “On the whole, I am satisfied with myself”). All items were rated on a 4-point Likert-type scale (1 = *Strongly disagree*, 4 = *Strongly agree*) and an overall score was computed by taking the mean of all items following reverse-coding of five items (higher scores reflect higher self-esteem). The scale has been shown to have a one-dimensional factor structure and good psychometric properties (e.g., Whiteside-Mansell and Corwyn, 2003). Cronbach's α for this measure was 0.77 at Time Point 1 and 0.87 at Time Point 2.

#### Paranormal beliefs

To measure paranormal beliefs and experiences, we used the Australian Sheep-Goat Scale (ASGS; Lange and Thalbourne, 2002; German translation: Voracek, 2009). In this 18-item scale, “sheep” refer to those who believe in the possibility of paranormal phenomena and “goats” refer to disbelievers of such phenomena (sample item: “I have had at least one experience of telepathy between myself and another person”). In its original form, the ASGS uses a visual analogue system for responses (Lange and Thalbourne, 2002). This response scale was modified to a 6-point Likert-type scale (0 = *Totally disagree*, 5 = *Totally agree*) by Voracek (2009), which was also the response format used in the present study. The scale is sometimes argued to consist of three subscales (relating to extrasensory perception, psychokinesis, and belief in the afterlife), but each of the subscales are highly inter-correlated and the preferred scoring has been to calculate an overall score as the mean of all items (for a discussion, see Voracek, 2009; higher scores on this scale reflect stronger belief in paranormal phenomena). Cronbach's α for the ASGS in the present study was 0.94 at Time Point 1 and 0.92 at Time Point 2, which is similar to values reported with previous use of the scale (Voracek, 2009; Swami et al., 2011).

#### Superstitious beliefs

Superstitious beliefs were measured using the 6-item Superstitious Beliefs Scale developed by Wiseman and Watt (2004; German translation: Voracek, 2009). The scale measures agreement with both negative (e.g., walking under a ladder) and positive (e.g., touching wood) superstitions on a 6-point Likert-type scale (0 = *Totally disagree*, 5 = *Totally agree*). Although separate scores can be calculated for negative and positive superstitious beliefs, Voracek (2009) recommended considering the scale one-dimensional and computing an overall superstitious beliefs score by taking the mean of all six items (higher scores on this scale reflect stronger superstitious beliefs). Cronbach's α for this scale was 0.86 at Time Point 1 and 0.79 at Time Point 2, which is similar to previous reports of the scale's internal consistency (Voracek, 2009; Swami et al., 2011).

#### Crystallized intelligence

To measure crystallized (general) intelligence, we included the *Wortschatztest* (WST) or German Vocabulary Test (Metzler and Schmidt, 1992; Schmidt and Metzler, 1992). This is a multiple-choice vocabulary test that comprises 42 items, each of which consists of one target word, interspersed among five made-up words (i.e., neologisms) as distractors. An overall score on the test consists of the sum of correctly answered items. The WST is often used for estimating cognitive functioning and is a good proxy for crystallized intelligence among German-speaking samples (Pietschnig et al., 2010). Furthermore, scores on the test correlate well with other measures of cognitive ability (e.g., Merten, 2005).

#### Media exposure

After Swami et al. (2010), we included a measure of exposure to conspiracist ideas about the kidnapping of Natascha Kampusch and the aftermath of the case. Participants rated the extent to which five items were true of themselves on a 9-point Likert-type scale (1 = *Completely false*, 9 = *Completely true*). The five items related to the extent to which participants had sought information about the Kampusch case from five distinct sources (the Internet, films or television programs, books or articles, friends or peers, and family members). Cronbach's α for the total scale was 0.59 at Time Point 1 and 0.57 at Time Point 2. However, item-total correlations were lower for the Internet (*r*_it_ = 0.28) and books or articles (*r*_it_ ≤ 0.17) than for the other three sources (*r*_it_ ranging between 0.31 and 0.59). Cronbach's α for the scale without the Internet and books or articles was 0.65 at Time Point 1 and 0.64 at Time Point 2. Thus, for analysis, we used scores of the three-item scale and the individual scores of the two remaining items.

#### Demographics

Participants provided their demographic details consisting of age, sex, ethnicity, and highest educational qualification.

### Procedure

Data collection was conducted using a snowball sampling technique. Specifically, several data collectors from the University of Vienna initiated sampling through their personal contacts, who in turn were requested to recruit further participants from their own networks. Potential participants were invited to complete a survey on their social and political opinions. The first testing period took place in October 2009 and the second testing period took place 2 years later in October 2011. Participants from both time points completed an identical survey in which the components of the survey were semi-randomized. All participants took part on a voluntary basis, were not remunerated for participation, and were provided with a debrief sheet once they had completed the survey.

## Results

### Dimensional analysis of conspiracist beliefs about Natascha Kampusch

In order to examine the factor structure of the KCS, we computed principal-axis exploratory factor analysis (EFA) using quartimax rotation (because of the expectation of the emergence of a general factor; Pedhazur and Schmelkin, 1991) using the data from Time Points 1 and 2 separately. The number of factors to be extracted was determined by factor eigenvalues (λ) above 1.0 (the EGV1 criterion) and based on the scree-plot criterion (Cattell, 1966). However, these techniques can lead to factor over-retention (Patil et al., 2010), so parallel analysis was also conducted. This technique generates eigenvalues from random datasets that match the actual dataset in terms of the number of participants and variables, and is considered a more accurate technique for determining the number of factors to retain from EFA (Hayton et al., 2004).

For the data from Time Point 1, Barlett's test of sphericity, χ^2^_(66)_ = 909.69, *p* < 0.001, and the KMO measure of sampling adequacy, KMO = 0.83, showed that the KCS items had adequate common variance for factor analysis. The EGV1 criterion and examination of the scree-plot suggested it was possible to extract three factors after six iterations. However, parallel analysis showed that only the first eigenvalue for the random data set (2.59) was smaller than its real data counterpart (2.64). By contrast, the second and third eigenvalues for the random data set were larger (2.44 and 1.93) than the second and third eigenvalues for the real data (2.39 and 1.82). These results indicate that only the first factor should be retained. Examination of the item loadings (see Table 1) indicated that 8 of the 12 items of the KCS loaded onto the first factor. This factor explained 22.0% of the variance. Further examination of the inter-correlations between the first and second factors indicated that these were strongly correlated (*r* = 0.69), suggesting substantial overlap in content coverage.

For the data from Time Point 2, Barlett's test of sphericity, χ^2^_(66)_ = 1177.34, *p* < 0.001, and the KMO measure of sampling adequacy, KMO = 0.88, again showed that the KCS items had adequate common variance for factor analysis. Based on the EGV1 criterion and examination of the scree-plot, it appeared that two factors could be extracted after four iterations. As before, however, parallel analysis showed that only the first eigenvalue for the random data set (2.62) was smaller than its real data counterpart (2.89), whereas the second eigenvalue for the random data set was larger (2.52) than the second eigenvalues for the real data (2.51). These results indicate that only the first factor should be retained. Item loadings (see Table 1) suggested a similar pattern of loadings as with the data from Time Point 1, with 8 of the 12 KCS items loading onto the first factor (explaining 24.1% of the variance). Furthermore, the first and second factors were highly inter-correlated (*r* = 0.67), suggesting content overlap between the two factors.

Based on the above results, we computed an overall score of conspiracist beliefs about the Natascha Kampusch case by taking the mean of the eight items that loaded on the first factor. The scale showed good internal consistency at both Time Point 1 (α = 0.78) and Time Point 2 (α = 0.83). Analysis of covariance (ANCOVA; factors: time point and sex; covariate: age) showed no significant sex differences in KCS scores at both time points, *F*_(1, 524)_ = 0.02, *p* = 0.897, η^2^_p_ < 0.01, no interaction of time point with sex, *F*_(1, 524)_ = 0.22, *p* = 0.640, η^2^_p_ < 0.01, and no effect of age on KCS scores, *F*_(1, 524)_ = 0.23, *p* = 0.634, η^2^_p_ < 0.01. However, there was a nominally significant difference in the strength of conspiracist beliefs about Kampusch between Time Point 1 (*M* = 3.72, *SE* = 0.09) and Time Point 2 (*M* = 3.45, *SE* = 0.10), although the effect size was small, *F*_(1, 524)_ = 3.98, *p* = 0.046, η^2^_p_ = 0.01. Further univariate analyses of variance (ANOVAs) revealed a significant difference between time points in self-esteem, although again the effect size was negligible. There were no other significant differences in included variables between time points (see Table 2). Notably, however, participants had sought information about the Kampusch case mostly from films and television, friends, peers, and family, but generally less from the Internet and books or articles.

**Table 2:** **Univariate analyses of variance for all dependent variables with time point as the independent variable**.

	**Time Point 1**		**Time Point 2**		***F***	***P***	**η^2^_*p*_**
	***M***	***SD***	***M***	***SD***			
Conspiracist ideation	3.88	1.44	3.83	1.37	0.14	0.709	<0.01
Self-esteem	3.46	0.43	3.34	0.52	8.53	0.004	0.02
Paranormal beliefs	2.47	1.10	2.38	0.98	1.03	0.311	<0.01
Superstitious beliefs	1.89	1.05	1.94	0.97	0.40	0.527	<0.01
Crystallized intelligence	31.05	5.52	30.90	5.81	0.09	0.765	<0.01
Media exposure to Kampusch conspiracies
Films or television programs, friends or peers, and family members	5.94	2.08	5.80	1.94	0.65	0.420	<0.01
Internet	1.96	2.15	2.22	2.24	1.87	0.172	<0.01
Books or articles	1.27	1.00	1.30	1.21	0.12	0.731	<0.01

### Personality and further correlates of conspiracist beliefs about Natascha Kampusch

We next computed bivariate correlations between belief in the Kampusch conspiracy theory and all remaining variables at Time Points 1 and 2, respectively. As can be seen in Table 3, at Time Point 1, stronger belief in the Kampusch conspiracy was significantly associated with greater conspiracist ideation, greater paranormal beliefs, greater superstitious beliefs, lower crystallized intelligence, and greater overall exposure to media about the Kampusch conspiracy, excluding, however, exposure to the Internet. The same pattern was replicated at Time Point 2, with the exception of the association between belief in the Kampusch conspiracy theory and crystallized intelligence, which no longer reached significance. In contrast to Time Point 1, greater belief in the Kampusch conspiracy correlated with greater exposure to the Internet at Time Point 2 but ceased to correlate with exposure to books or articles. Moreover, having sought information from books or articles was negatively correlated with self-esteem at Time Point 1 but not at Time Point 2.

**Table 3:** **Inter-scale bivariate correlations between belief in the Kampusch conspiracy theory and all remaining variables**.

	**(1)**	**(2)**	**(3)**	**(4)**	**(5)**	**(6)**	**(7)**	**(8)**	**(9)**
(1) Belief in Kampusch conspiracy		0.56^**^	−0.11	0.32^**^	0.14^*^	−0.12^*^	0.21^**^	0.03	0.18^**^
(2) Conspiracist ideation	0.59^**^		−0.12^*^	0.37^**^	0.12^*^	−0.19^*^	0.14^*^	0.06	0.12
(3) Self-esteem	−0.06	−0.10		0.05	−0.07	0.05	0.03	−0.08	−0.28^**^
(4) Paranormal beliefs	0.28^**^	0.49^**^	−0.04		0.26^**^	−0.23^**^	0.11	−0.08	−0.01
(5) Superstitious beliefs	0.20^**^	0.21^**^	0.07	0.37^**^		−0.19^*^	0.28^**^	0.08	0.10
(6) Crystallized intelligence	−0.07	−0.18^*^	−0.03	−0.19^*^	−0.21^**^		−0.09	0.11	−0.05
(7) Media exposure	0.23^**^	0.24^**^	−0.01	0.19^**^	0.27^**^	−0.16^*^		0.25^**^	0.01
(8) Internet	0.16^*^	0.12	−0.09	0.04	0.03	−0.08	0.18^**^		0.22^**^
(9) Books or articles	0.01	0.11	0.01	0.12^*^	0.08	−0.02	0.15^*^	0.13^*^	

Note:

* p < 0.01,

** *p < 0.001*.

Time Point 1 = above diagonal, Time Point 2 = below diagonal.

We also computed a multiple linear regression model with belief in the Kampusch conspiracy as the criterion variable and time point, sex and age, and all other remaining variables entered simultaneously as predictors. Furthermore, we also added interaction terms of time point with each predictor to the regression model to account for possible differences between time points. The regression model was significant, *F*_(21, 504)_ = 15.43, *p* < 0.001, Adj. *R*^2^ = 0.37, with conspiracist ideation (*B* = 0.48, *SE* = 0.05, β = 0.47, *t* = 9.01, *p* < 0.001), media exposure (three-item scale; *B* = 0.09, *SE* = 0.04, β = 0.13, *t* = 2.59, *p* = 0.010), exposure to books or articles (*B* = 0.18, *SE* = 0.07, β = 0.14, *t* = 2.48, *p* = 0.014), and paranormal beliefs (*B* = 0.15, *SE* = 0.07, β = 0.11, *t* = 2.15, *p* = 0.032) emerging as significant predictors. Notably, interactions of exposure to books or articles with time point (*B* = −0.29, *SE* = 0.10, β = −0.21, *t* = −2.95, *p* = 0.003) and of exposure to the Internet with time point (*B* = 0.12, *SE* = 0.05, β = 0.15, *t* = 2.35, *p* = 0.019) also turned out significant. I.e., exposure to books or articles lost its predictive value at Time Point 2, whereas exposure to the Internet gained predictive value at Time Point 2. None of the other predictors or interactions were significant (*p*s ≥ 0.098).

## Discussion

In the present study, we examined the temporal stability of belief in conspiracy theories concerning the kidnapping case of Natascha Kampusch. Our results indicated, firstly that those beliefs could be reduced to a single dimension that reflected a general conspiracist point of view concerning the Kampusch case. It is worth noting that, although our measure included a number of disparate claims (e.g., that Kampusch's parents sold her into captivity and that investigators were covering up evidence of multiple perpetrators), it was apparent from our factor analysis that the items in our measure reduced to a single dimension at both time points. This suggests that conspiracist ideas surrounding the Kampusch case can best be considered as generally one-dimensional.

In addition, we examined the temporal stability of those conspiracist ideas across a 2-year period beginning in October 2009. The first thing to note is that mean scores at both time points were below the mid-point of the scale, thus suggesting relatively low-level agreement with the items was typical. Moreover, the strength of those beliefs remained relatively stable over the period of examination. Although there was a significant decline in the strength of belief in the conspiracy theory between Time Points 1 and 2, the effect size of the difference was very small and therefore of little practical significance. This is in tandem with our finding that the strength of belief in general conspiracy theories did not differ significantly over testing periods.

Both Gray (2008) and Swami (2012) have argued that, when examining particular conspiracy theories within a specific national context, it will be important to consider the way in which local socio-political, economic, and cultural factors affect conspiracist ideation. Here, using a repeated cross-sectional design, we were able to show that belief in a conspiracy theory relatively unique to Austria remained stable over a period of 2 years. Although we cannot conclusively link this with stability in the wider Austrian context during the same period, it seems likely that conspiracy theories concerning the Kampusch case reached saturation by 2009 and that belief in those theories has remained relatively stable since, albeit at a generally low level.

Consistent with previous work (Goertzel, 1994; Swami et al., 2010, 2011; Swami and Furnham, 2012; Wood et al., 2012), our results also showed that belief in the Kampusch conspiracy theory at both time points was significantly predicted by a general conspiracist worldview, or belief in other conspiracy theories. This is consistent with the suggestion that conspiracy theories form part of a monological belief system, wherein a conspiracist worldview eases the assimilation of new conspiracy theories (Goertzel, 1994). In this way, conspiracy theorists are able to assimilate explanations for novel phenomena and events that may otherwise be difficult to understand or that would threaten prevailing worldviews.

Our results also showed that belief in the Kampusch conspiracy theory was relatively weakly but significantly associated with media exposure to the conspiracy at both time points. Moreover, we obtained evidence of changing effects with regard to specific sources of information over time even though media exposure itself remained stable. Effects of films or television programs, of friends or peers, and of family remained stable over time, suggesting a stable role of these sources with regard to the formation and maintenance of belief in the Kampusch conspiracy. However, we observed a switch in the effects of print media and the Internet. Print media fuelled belief in the Kampusch conspiracy at Time Point 1 but not Time Point 2, whereas the Internet had no influence on the belief at Time Point 1 but gained influence at Time Point 2. These findings suggest that the dissemination of information sustaining the Kampusch conspiracy may have declined in print media between 2009 and 2011. Conversely, the circulation of conspiracy theories on the Kampusch kidnapping on the Internet appears to have increased by 2011. However, importantly, these changes did neither increase nor decrease overall levels of belief in the Kampusch conspiracy between 2009 and 2011. This corroborates again that conspiracy theories may be part of a monological belief system. Finally, of note and with the exception of paranormal beliefs in Time 1, none of the other variables included in our study emerged as significant predictors of belief in the Kampusch conspiracy.

The main limitation of the present study concerns the use of a repeated cross-sectional design. Although this method has its advantages (Moffit, 1993; Verbeek and Vella, 2005), it limits the conclusions that can be drawn on the basis of the existing dataset. For example, although it is possible to use a repeated cross-sectional design to examine the extent to which belief in a particular conspiracy theory changed between two time points, to assess whether a person who subscribed to the conspiracy theory at Time Point 1 is more or less likely to believe in the theory at Time Point 2 requires the use of true longitudinal data. A longitudinal design also has other benefits over a repeated cross-sectional design, including increased statistical power and the potential to estimate a greater range of conditional probabilities.

In a similar vein, it is possible that differences in the cross-section of participants in each time point affected the nature of our results. That is, it is not possible to say that the samples recruited at either time point were representative of the wider Austrian population, thus limiting the generalizability of our findings. Furthermore, the use a snowball sampling technique likely resulted in a biased sample. For instance, participants who had been debriefed about the study may have selectively recruited other participants. It is therefore not possible to establish with certainty the background level of conspiracist belief, although any bias will likely have been preserved across sampling periods.

Other limitations of our work include the relatively small sample sizes at each time point and the limited number of additional variables included in our design. In terms of the latter in particular, we did not include any variables that might predict change in conspiracist beliefs, which should be considered in future work. Nevertheless, the present work begins the important task of examining the temporal stability of conspiracy theories over time. The use of true longitudinal datasets will undoubtedly assist scholars in this area of research to understand the nature of conspiracy theories in greater detail. Highlighting points at which specific conspiracy theories change and/or become relatively stable within a given population may assist scholars in understanding the transmission of conspiracy theories within a given population.

### Conflict of interest statement

The authors declare that the research was conducted in the absence of any commercial or financial relationships that could be construed as a potential conflict of interest.
